# Supplementary material for: Structural basis for selective thymidine binding by the Borrelia burgdorferi substrate-binding protein BmpA
Source: J Biol Chem. 2026 May 27;302(7):113206. doi: 10.1016/j.jbc.2026.113206 (PMC13325309; doi:10.1016/j.jbc.2026.113206)
Supplement: Supporting Figures [file mmc1.docx]

**Supporting Information Available**

**
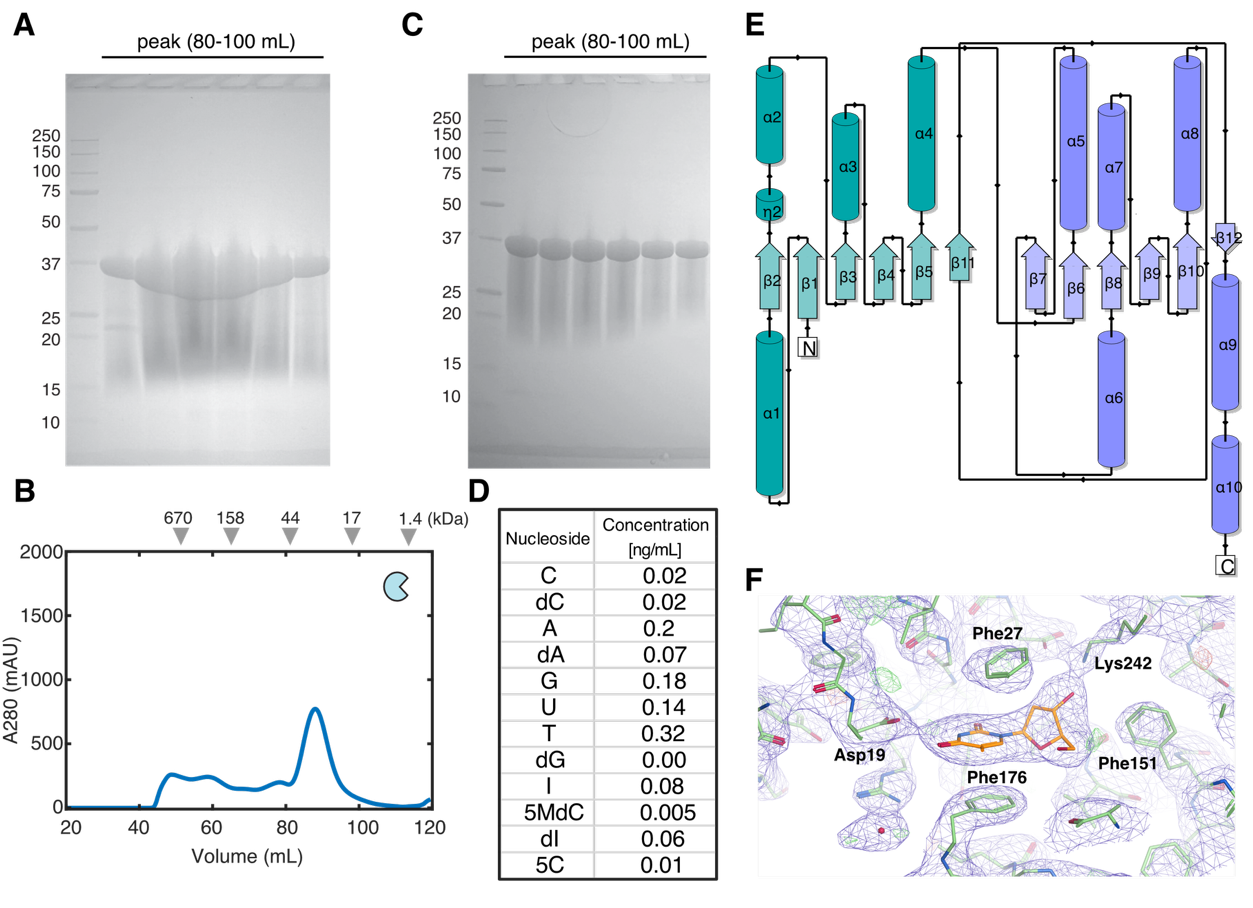
**

**Figure S1: A.** SDS-PAGE of purified BmpA protein. **B.** SEC trace of unfolding/refolding treated BmpA protein. **C.** SDS-PAGE of unfolding/refolding treated BmpA protein. **D.** LC-MS analysis of unfolding/refolding treated BmpA. **E.** Schematic of BmpA secondary structure. The N-terminal domain is colored teal, and the C-terminal domain is purple. **F.** Electron density maps at the binding site. The 2Fo-Fc map (1.3σ , purple mesh) and Fo-Fc map (4.0σ, green and red mesh) are shown around the ligand (thymidine, orange sticks) and surrounding protein residues (green sticks).


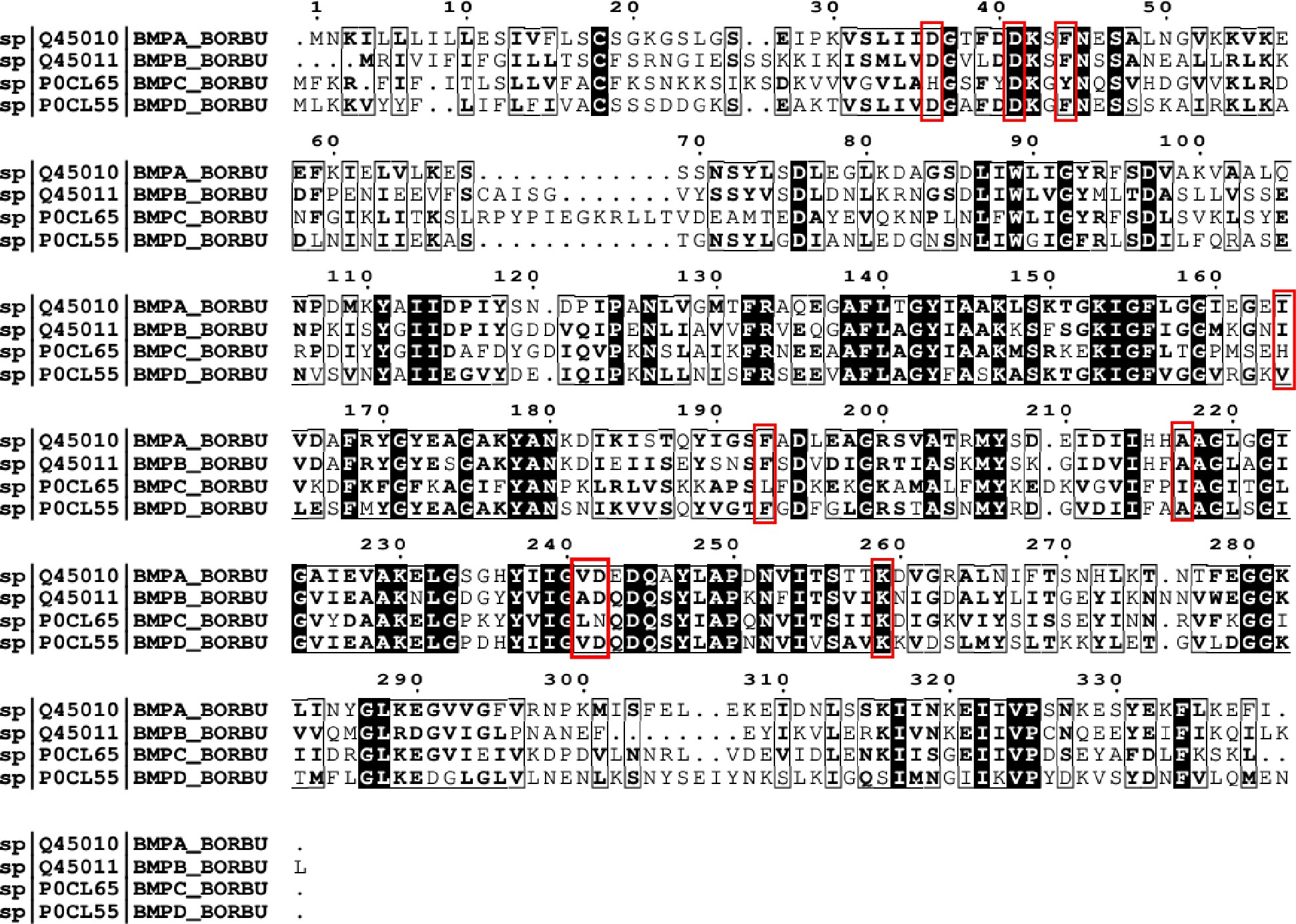


**Figure S2:** Sequence alignment of BmpA with BmpB, BmpC and BmpD. White characters in a black box indicate strict identity, bolded characters indicate similarity within a group, a black frame indicates similarity across groups, and residues involved in ligand contact are boxed in red. Residue numbering is based on BmpA.


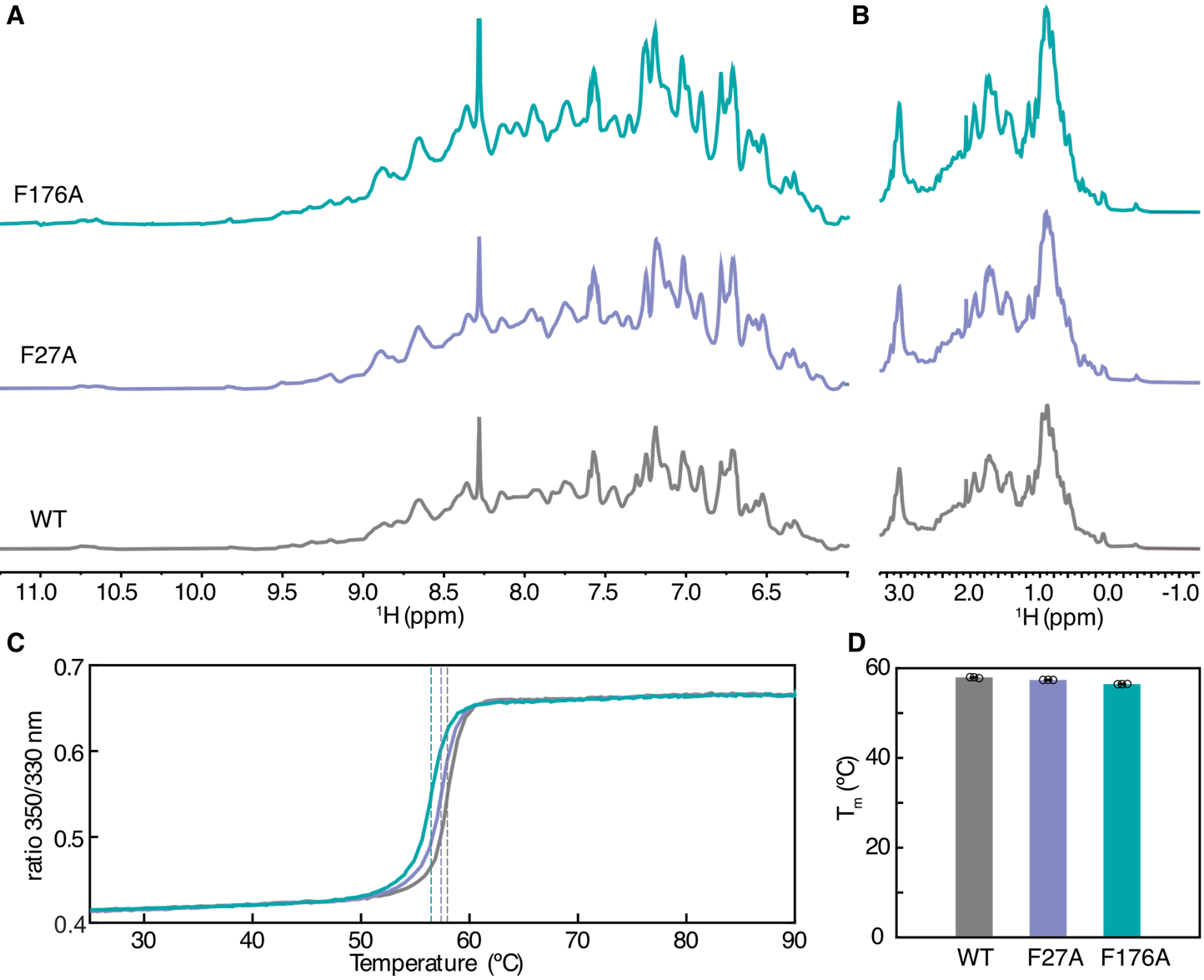


**Figure S3:** Structural and thermal comparison between BmpA and our variants. **A. and B.** Downfield **(A)** and upfield **(B)** proton NMR spectra displaying a similar and wide peak dispersion between BmpA and the alanine substitution variants. **C.** NanoDSF thermal unfolding curves from 25 °C to 90 °C for BmpA and the variants. Dashed lines denote the melting temperature for each species. **D.** Melting temperatures determined with nanoDSF. Data are presented as mean ± SD (n=three independent measurements, open circles). No significant differences were observed between groups (one-way ANOVA, p > 0.05).
